# Cause of Death Analysis in a 9½-Year-Old with COVID-19 and Dravet Syndrome

**DOI:** 10.3390/pathophysiology32010003

**Published:** 2025-01-10

**Authors:** Vedashree R. Meher, Richard J. Huntsman, Francis H. Y. Green, Jill C. Wooff, Roland N. Auer

**Affiliations:** 1 Division of Anatomical Pathology, Department of Pathology, College of Medicine, University of Saskatchewan, Royal University Hospital, 103 Hospital Drive, Saskatoon, SK S7N 0W8, Canada; roland.auer@usask.ca; 2 Division of Pediatric Neurology, IWK Health Centre, 5850/5980 University Avenue, Halifax, NS B3K 6R8, Canada; rhuntsman@dal.ca; 3 Department of Pathology, University of Calgary, Foothills Medical Centre—McCaig Tower, 3330 Hospital Drive NW, Calgary, AB S7N 0W8, Canada; fgreen@ucalgary.ca; 4 Department of Pathology, Pasqua Hospital, 4101 Dewdney Avenue, Regina, SK S4T 1A5, Canada; jill.wooff@saskhealthauthority.ca

**Keywords:** Dravet syndrome, epilepsy, seizure, sudden unexpected death in epileptic patients, SUDEP, COVID-19, cellular interstitial pneumonia, virus, co-pathology

## Abstract

**Background**: Cause of death analysis is fundamental to forensic pathology. We present the case of a 9½-year-old girl with a genetically confirmed diagnosis of Dravet syndrome who died in her sleep with no evidence of motor seizure. She also had a lifelong history of recurrent pneumonias and, along with her family, had tested positive for COVID-19 10 days before death. **Methods**: Long-term clinical history of Dravet Syndrome and respiratory infections were obtained from patient’s medical charts and radiology reports. A Rapid-Antigen Test was used to confirm SARS-CoV2 infection days prior to death. At autopsy, brain, heart and lung tissues were obtained. Paraffin-embedded tissues were double-stained with H&E, and immunohistochemically stained using various antibodies. **Results**: Autopsy revealed evidence of previous seizure activity in the brain and cellular interstitial thickening in the lung. The brain showed edema and fibrillary gliosis without neuronal loss in neocortex and hippocampus. The lung showed inflammatory interstitial thickening with histiocytes, megakaryocytes, B-lymphocytes, and T-lymphocytes, including helper/suppressor cells and cytotoxic T-lymphocytes. Diffuse alveolar damage was observed as alveolar flooding with proteinaceous fluid. **Conclusions**: The cause of death may be attributed to Sudden Unexpected Death in Epilepsy (SUDEP) in Dravet syndrome, sudden death in viral pneumonia, or some combination of the two. When two independent risk factors for sudden unexpected death are identified due to co-pathology, it may not be possible to determine a single cause of death beyond a reasonable doubt.

## 1. Introduction

Analysis of the cause of death is an essential part of forensic pathology. Death ultimately results from the cessation of both cardiac and pulmonary functions, with cardiovascular and pulmonary diseases being a major contributing factor of death. However, the penultimate cause of death may lie in the brain, such as seen in epilepsy, since brain dysfunction can lead to the cessation of cardiopulmonary function and death in some circumstances. Pulmonary dysfunction can also lead to death by causing a cardiopulmonary arrest, without brain dysfunction.

We report the case of a 9½-year-old girl with Dravet syndrome who died in her sleep. In addition to mild brain damage consistent with long-standing epilepsy, the autopsy showed cellular interstitial pneumonia related to SARS-CoV-2 infection.

Dravet syndrome (DS) was described by Charlotte Dravet in 1978. It is also known as Severe Myoclonic Epilepsy of Infancy (SMEI) and is caused by *de novo* SCN1A gene mutations in over 85% of patients [1]. It is usually fatal by age 10 [2].

The COVID-19 pandemic is caused by a coronavirus that enters pneumocytes via a receptor for angiotensin-converting enzyme 2 (ACE2), resulting in a cellular interstitial pneumonitis [3,4,5,6], which can also be fatal.

We present a case report focusing on the pathophysiological analysis of a child’s death who had a long-term history of both epilepsy and respiratory infections where the precise cause of death is unclear.

## 2. Materials and Methods

### 2.1. Clinical History

#### 2.1.1. Neurological History

The patient’s neurological symptoms began in early infancy with recurrent episodes of febrile status epilepticus, characterized by prolonged hemi-convulsive seizures followed by post-ictal Todd’s paralysis. In addition, the patient had congenital thoracolumbar scoliosis with convexity to the left. Brain CT and MRI at ages 5 and 6.5 months showed prominent bilateral extra-axial spaces without evidence of atrophy, hydrocephalus, acute intracranial hemorrhages, or mass effect.

These seizures failed to respond to conventional anticonvulsants, including phenobarbital. Once a diagnosis of DS was genetically confirmed, treatments including valproate, stiripentol, clobazam, and ketogenic diet were initiated, together with carnitine for hepatic protection and ketosis augmentation. Follow-up brain CT performed at 20 months of age showed no atrophy or radiographic evidence of hydrocephalus.

By the age of 2, she exhibited cognitive delays, functioning globally at the level typical for a 12–18 month old. Additionally, the patient experienced diffuse ataxia. The benefit from the ketogenic diet waned, and she was weaned off valproate due to thrombocytopenia. However, after the clobazam dose was increased, the patient experienced 8 seizures per month, typically generalized tonic–clonic seizures lasting 1–2 min.

In May 2017, at age 5, she was enrolled in the Cannabidiol in Children with Refractory Epileptic Encephalopathy (CARE-E) study, receiving an escalating dose of 1:20 THC:CBD cannabis extract over 6 months to a target dose of 10–12 mg/CBD/kg/day. She tolerated the CBD-enriched cannabis extract well. There was a 40% reduction in seizures during the course of the study, and her parents reported the patient was brighter, more interactive, and showed improvement in expressive communication. However, the benefits waned, and CBD-enriched cannabis extract was discontinued at age 8.

A vagal nerve stimulator was implanted at age 8, with a regular output setting of 2.25 milliamps. At age 8, the patient weighed 22.3 kg (3rd–15th percentile) and was 121 cm (3rd–15th percentile) in length. She continued to exhibit developmental delays, functioning at approximately the level of a two-year-old, and continued to have frequent, approximately 50 per month, tonic–clonic seizures. Stiripentol was increased to a high dose of 87 mg/kg/day. Combined with the vagal nerve stimulation, this seemed to decrease her seizure frequency, but it also caused decreased appetite and weight loss. Consequently, levetiracetam was added up to 50 mg/kg/day. Her vagal nerve stimulator was adjusted to 2.5 milliamps, and she had 4 days without a seizure.

The patient slept well most nights unless she was actively seizing. She had frequent regression of mobility and communication with increased severity and frequency of seizures. Occasionally, she exhibited violent behavior and bit her tongue.

#### 2.1.2. Pulmonary History

A lifetime history of recurrent pulmonary infections began at age 1½. At 13 months of age, the patient was admitted for cough and seizures. Chest X-rays showed lower lung volumes and mild central bronchial wall thickening. At 20 months of age, the patient was admitted for recurrent seizures and respiratory distress. Chest X-rays showed increased radiopacity and indistinctness in right perihilar and right upper lobe with early infiltrates. There was extensive lobar consolidation of the right upper and middle lobes, with less extensive changes in the lower lobe, indicating radiographic evidence of pneumonia. From age 2 until age 3½, the patient had several hospital visits for fever, shortness of breath, wheezing, and cough. A near death episode of pneumonia occurred at age 3½, requiring hospitalization and intubation. Chest X-rays showed prominent bilateral interstitial markings, hazy opacities, and diffuse pulmonary nodules.

Ten days before her death, 3 family members tested positive for SARS-CoV-2 (COVID-19) including the patient, her older sibling, and her mother. SARS-CoV-2 infection was confirmed through a nasal swab and Rapid Antigen Test results. The patient’s exhibited symptoms of mild fever, fatigue, and weakness.

#### 2.1.3. ER Visit and Death

On the date of death, the parents went to check on the patient in the morning. Patient was found in the bedroom in a prone position and cyanotic. The sheets were undisturbed, with no vomitus, blood, or other staining. CPR was performed for 1 h 10 min, with 5 rounds of epinephrine and 4 rounds of atropine. Upon arrival to the emergency department, the respiratory therapist reported bilateral crackles to auscultation but good air entry with bagging. The patient was hypothermic, with a temperature of 34.6 °C, and resuscitation was not possible.

#### 2.1.4. Autopsy

With written parental consent, an autopsy was performed, with examination of the heart, lungs, and brain.

#### 2.1.5. Histology and Immunohistochemistry

All tissues were fixed in 10% buffered formalin and embedded in paraffin. Sections of 5 µm thickness were cut and placed on electromagnetically charged glass slides. All sections were routinely stained with Hematoxylin and Eosin (H&E).

Immunohistochemistry was performed to detect specific cellular markers including macrophages (CD68, Vimentin), megakaryocytes (CD61), B-lymphocytes (CD20), T-lymphocytes (CD8, CD4, and CD3), and glial cells (GFAP). Prior to staining, sections were deparaffinized in xylene and rehydrated in ethanol and water. To unmask antigens, sections underwent heat-induced epitope retrieval (HIER).

After the HIER procedure, sections were blocked using a peroxidase-blocking reagent to prevent non-specific binding. After application of primary antibody and horseradish-peroxidase-conjugated secondary antibody, sections were applied with chromogenic substrates to visualize antibody–antigen complexes. Finally, the sections were counterstained with hematoxylin and mounted using an aqueous mounting medium.

Light microscope images were captured at a resolution of 600 dpi and 300 dpi using Nixon microscope.

## 3. Results

### 3.1. Neuropathology

**Figure 1:**
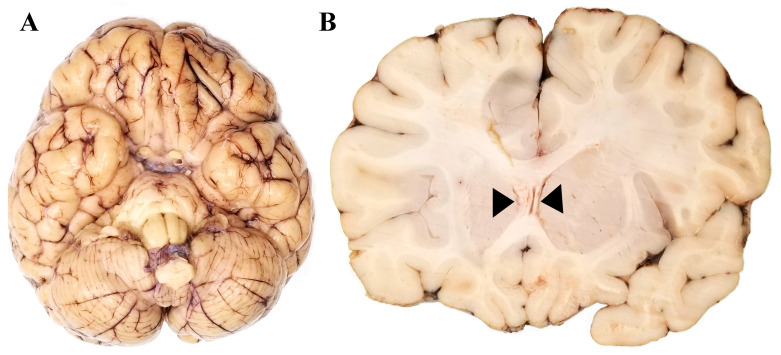
Neuropathological abnormalities in 9½-year-old patient with Dravet syndrome. (**A**). Gross examination reveals the brain to be small, in keeping with global developmental delay, with no atrophy of the cerebellum. (**B**). Macroscopic view of the coronal section shows edema, which is evidenced by compressed lateral ventricles (black arrowhead) as well as swollen and flattened gyri.

The brain grossly was small and round in configuration when viewed from above (Figure 1A), consistent with profound developmental delay. The cerebellum showed no specific atrophy but was small, in keeping with the global brain size. With no focal abnormalities externally, the brain nevertheless appeared mildly edematous. The unfixed brain weight was 1173 g (N = 1275–1290 g at this age) [7].

During brain dissection, the impression of brain edema was confirmed, with slit-like ventricles and the complete obliteration of sulci shown by swollen gyri over the cerebral convexities (Figure 1B). 

Blocks of tissue were collected from neocortex, white matter, insula, hippocampus, basal ganglia, thalamus, cerebellum, midbrain, pons, and medulla.

Brain tissue samples were stained with hematoxylin & eosin (H&E) and immunohistochemically with antibodies against glial fibrillary acidic protein (GFAP) and macrophages (CD68).

**Figure 2:**
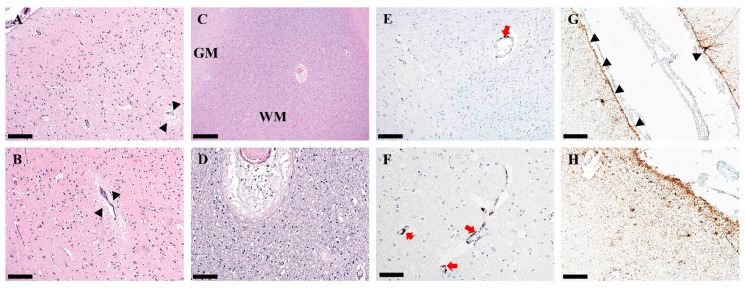
Histological changes in gray and white matter of the cortex in a 9½-year-old patient with Dravet syndrome. (**A**,**B**). Neuropil vacuolization of gray matter edema is seen, especially around blood vessels (arrowheads). (**C**,**D**). Lower magnification shows gray matter (GM) and white matter (WM) of the neocortex (**C**), and higher magnification shows fiber-splitting around blood vessel, indicative of white matter edema (**D**). (**E**,**F**). CD68-positive microglia are seen infiltrating the perivascular space (red arrows). (**G**,**H**). GFAP-positive fibrillated astrocytes are seen under the pia mater (arrowheads) as well as layers I and II of the frontal cortex (**H**). Scale bars—(**A**,**B**,**E**) magnification 10×. Bars = 200 µm, (**C**) magnification 4×. Bar = 500 µm and (**D**,**F**–**H**) magnification 20×. Bars = 100 µm.

Sections of left and right frontal lobe, parietal lobe, occipital lobe, temporal lobe, and insula all showed similar changes (Figure 2). There was evidence of edema of both gray and white matter. The gray matter edema manifested as a vacuolization of the neuropil, often clustered around blood vessels (Figure 2A,B). Fiber-splitting white matter edema was seen, often with perivascular edema (Figure 2C,D). A few microglial rod cells were seen in increased numbers, and immunohistochemistry for CD68 revealed numerous perivascular infiltrating microglia/macrophages (Figure 2E,F). Chaslin’s sub-pial gliosis was seen as densely fibrillated astrocytes on GFAP immunohistochemistry beneath the pia mater and extending into cortical layers I and II. Some perivascular GFAP was observed in the perivascular glia limitans (Figure 2G,H).

**Figure 3:**
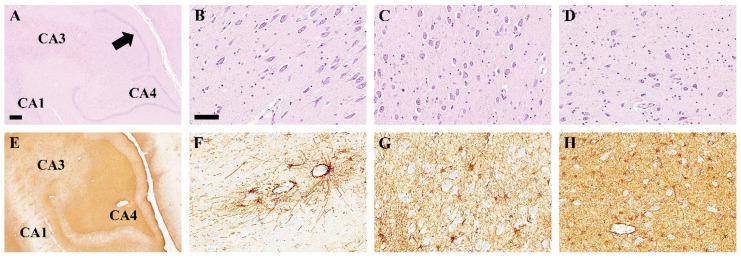
Histological changes in the hippocampus of a 9½-year-old patient with Dravet syndrome. (**A**–**D**). H&E-stained sections show no neuronal loss in the dentate gyrus (arrow), CA1 (**B**), CA3 (**C**), and CA4 regions (**D**–**H**). GFAP immunohistochemistry shows hippocampal astrogliosis, with minimal gray matter gliosis in CA1 (**F**) compared to CA3 (**G**) and CA4 regions (**H**). Scale bar—(**A**,**E**) magnification 4×. Bars = 1 mm, (**B**–**D**) and (**F**–**H**) magnification 20×. Bars = 100 µm.

The left hippocampus and the right hippocampus showed identical pathological changes (Figure 3). Granule cells in the dentate gyrus showed no dispersion, and there was no neuronal loss in the dentate gyrus or the pyramidal zones of CA4, CA3, or CA1 (Figure 3A–D). However, significant fibrillary astrogliosis was revealed on GFAP immunohistochemistry. Gray matter gliosis was dense in the above neuronal sectors of the hippocampus, with CA1 being relatively spared, showing minimal fibrillary gliosis (Figure 3E–H).

### 3.2. Pulmonary Pathology

The examination of the respiratory system revealed bilateral serosanguinous pleural effusions, pulmonary edema, and congestion. Right/left lung weights were 322/278 g, respectively (Normal R/L = 174/152 g to 177/166 g) [7]. There was no evidence of pulmonary embolism.

Lung samples were stained with H&E and Martius Scarlet Blue (MSB) stains and immunohistochemically with antibodies against macrophages (CD68), T-lymphocytes (CD3), T-helper/suppressor lymphocytes (CD4), cytotoxic T-lymphocytes (CD8), B-lymphocytes (CD20), megakaryocytes (CD61), and vimentin.

**Figure 4:**
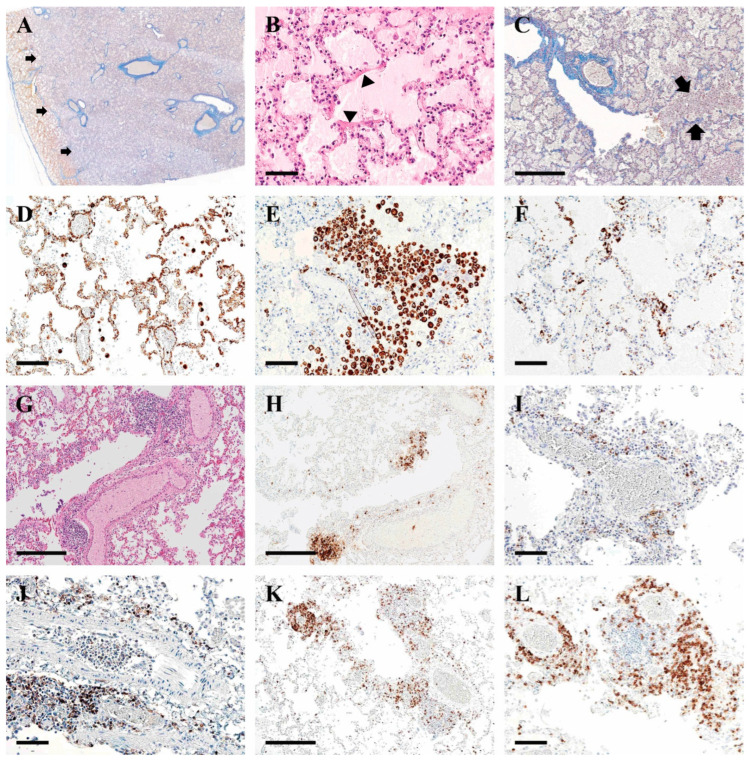
Histological changes in the lungs of a 9½-year-old patient with Dravet syndrome positive for COVID-19. (**A**). Martius Scarlet Blue (MSB)-stained section shows a section of the lung at 1× magnification, highlighting a sharp demarcation (arrows) between aerated (at left) and flooded airspaces (at right) (**B**). H&E-stained section shows alveolar edema with features of proteinosis and hyaline membranes (arrowheads). (**C**). Magnification of MSB image shows infiltration of macrophages into the bronchiole and alveolar ducts (arrows). (**D**). Vimentin immunohistochemistry shows activated macrophages, especially in the alveoli. (**E**). CD68-positive macrophages are seen in clumps and individually scattered. Some are binucleated (**E**,**F**). CD61-positive megakaryocytes are seen individually as well as clustered in groups. (**G**,**H**). B-lymphocytes are seen on H&E (**G**) and CD20 (**H**) forming perivascular aggregates and individually scattered throughout the interstitium. (**I**,**J**). CD8 cytotoxic T-lymphocytes (**I**) and CD4-positive T-helper/suppressor cells (**J**) are scattered throughout the lung and form perivascular aggregates (arrowheads) (**K**,**L**). CD3 T-cells follow a similar perivascular and peribronchiolar distribution. Scale bars—(**C**,**G**,**H**,**K**) magnification 10×. Bars = 300 µm and (**B**,**D**–**F**,**I**,**J**,**L**) magnification 20×. Bars = 100 µm.

The MSB stained sections showed distinct zones of alveolar edema juxtaposed with a zone of aerated alveoli (black arrows) (Figure 4A). The alveolar edema manifested as dense proteinaceous, eosinophilic fluid within the alveoli. In some areas, the edema showed artifactual cracks characteristics of alveolar proteinosis. Occasional hyaline membranes were seen at the junctions of the alveolar ducts with the alveoli, indicative of acute lung injury (black arrowheads) (Figure 4B). There was also a cellular interstitial pneumonitis with moderate alveolar type II cell hyperplasia, indicative of active repair (Figure 4B).

The alveoli contained large numbers of alveolar macrophages, particularly at the junctions connecting the alveolar ducts with the alveoli (arrows) (Figure 4C).

Immunohistochemical stains for inflammatory cell subtypes revealed vimentin-positive macrophages, with strong cytoplasmic positivity, indicating macrophage activation [8] (Figure 4D). CD68 staining revealed macrophages distributed throughout the interstitium, both in clumps and individually (Figure 4E). CD61 staining showed numerous individual and clustered megakaryocytes (Figure 4F). Lymphocytic infiltrates were observed throughout the lungs. CD20-positive B-lymphocytes formed nodules around blood vessels, rather than in their usual peribronchiolar location. Several clusters of B-lymphocytes accompanied vessels, typically arterioles but sometimes venules. Individual B-lymphocytes were also scattered throughout the lung parenchyma (Figure 4G,H).

T-lymphocytes were widespread, mostly as individual cells but also in perivascular and peribronchiolar clumps. CD4 and CD8 immunohistochemistry revealed many T-helper cells and cytotoxic T-suppressor cells (Figure 4I–L).

No intravascular thromboses was seen in the pulmonary vasculature. There was no interstitial fibrosis and no evidence of secondary bacterial bronchopneumonia, such as the neutrophilic infiltration of alveoli or bronchi.

### 3.3. Heart

The heart weighed 138 g (N = 115–116 g) [7]. The pericardial cavity contained serous fluid, but no other cardiovascular abnormalities were observed. The microscopic examination of the heart sections revealed no abnormalities. The enlargement of the heart was the only anomaly observed. Cardiac loading accompanying the increased cardiac output during seizures may account for the increased heart weight.

## 4. Discussion

Death necessarily involves the ultimate cessation of both cardiac and pulmonary function. A critical question in this case is whether some form of penultimate brain dysfunction preceded the cessation of cardio-pulmonary function. This may be relevant to other cause-of-death analyses in forensic science. With this question in mind, we first review DS and COVID-19, each in isolation. We then address potential interactions between the components of co-pathology in this case and, generally, in analyzing deaths.

### 4.1. Dravet Syndrome

The SCN1A gene encodes the α1 subunit of the voltage-gated sodium channel in the neuronal cell membrane, causing a loss of function in the sodium channel of inhibitory GABAergic interneurons while having no effect on the electrophysiological properties of pyramidal neurons [9]. Most children with DS have the onset of seizures in the first year of life, typically triggered by a febrile illness or sometimes following a vaccination. The initial seizures are often prolonged hemi-clonic convulsions. As the child gets older, other seizure types, including generalized myoclonic and intractable tonic–clonic seizures, predominate. Seizures are often triggered by fever, but frequently no trigger is found.

Developmental delay becomes apparent within two years of life, with most children having severe intellectual disability. Up to 40% of children with DS will be diagnosed with autism spectrum disorder, with 13% of patients being non-verbal [10].

The risk of death is 13 times higher in children with Dravet syndrome compared to age-matched healthy peers. The risk of mortality reaches 15% by 17 years of age. The most common causes of death in DS are SUDEP, or Sudden Unexpected Death in Epilepsy, and status epilepticus [10]. In a recent large series of 100 patients with Dravet syndrome having a median follow-up of 17 years, the median age at death was 7 years in the 17 patients who died [11].

Another important factor to consider is the effect of SCN1A gene mutation on the respiratory centers. An animal study by Kuo et al. showed that mutations in the SCN1A gene can cause hypoventilation, apnea, and diminished ventilatory response to carbon-dioxide, thereby contributing to a high incidence of SUDEP among DS patients [12].

### 4.2. Seizures and Neuronal Loss

Seizures do not always cause neuronal loss [13]. Neuronal death due to a pure epileptic insult, without hypoxia, requires uninterrupted seizure activity of approximately 1 h [14]. Our patient had frequent seizures, but they lasted for about 1 to 2 min. This may explain the intact neocortical and hippocampal neuronal populations seen here in particular and the paucity of neuropathologic findings in DS in general [15]. Gliosis is a more sensitive structural indicator of a CNS insult than neuronal loss. This is borne out by the present case and generally with mild brain insults such as spreading depolarization (previously termed spreading depression) [16].

Seizures that are tonic in the diaphragm are more likely to cause death than clonic seizures due to the arrest of breathing with a tonus of the diaphragm. The lack of ruffling or staining of the bedsheets argues for tonic, rather than clonic, seizure activity if a seizure indeed occurred and was the cause of death.

The lack of recent status epilepticus, aspiration, or accident would render the cause of death as SUDEP. SUDEP is more common in DS than in other forms of epilepsy [17]: in a study of 100 Dravet syndrome patients, the Dravet-specific SUDEP rate was 9.32/1000 person-years, with deaths mostly in childhood, compared to a 5.1 SUDEP rate/1000 person-years for refractory epilepsy in adults [11].

### 4.3. COVID-19

Hypoxia can cause cardiac arrest [18,19], and hypoxia is the most common premonitory sign preceding cardiac arrest in SARS-CoV-2 infection [20,21,22]. Children have lower mortality rates from COVID-19 compared to adults due to differences in humoral immune responses [23] and ACE2 receptor expression, which increases with age [24]. A study by Yonker et al. showed that the expression of ACE2 cannot solely explain pathogenicity because children can carry high viral loads despite low ACE2 expression levels.

In deaths due to COVID-19, reports have found neocortical neuronal loss and perivascular brain lymphocytes [25], but this patient’s brain showed only fibrillary astrogliosis, attributable to the chronic seizures.

The pulmonary histological features of COVID-19 seen in this patient are similar to those reported in other studies of fatal cases [6,26]: intra-alveolar macrophages, some binucleate, capillary congestion, the formation of fibrinous hyaline membranes, diffuse alveolar damage, and type II pneumocyte hyperplasia. Interstitial lymphocytic infiltrates of B and T lymphocytes were detected throughout the lung. Some severe COVID-19 cases have also reported the development of pulmonary alveolar proteinosis [27,28,29,30,31]. In this case, there was severe alveolar edema with some features of proteinosis. We found no areas of organizing pneumonia, which suggested that there was no progression to the fibrotic phase of DAD as noted in some studies [32,33]. This was likely due to a shorter infection duration of only 10 days in this case.

Immunohistochemically, the CD61-positive pulmonary megakaryocytes can be attributed to hypoxia [34], lung inflammation [35,36], or immunity [37]. The increased presence of CD61-positive cells in COVID-19 patients with DAD indicates a prothrombotic response and may contribute to fibrosis [38]. Vasculopathy has been commonly associated with COVID-19 [33] as a vascular response to the inflammation of the tunica intima and prothrombotic transformation [39], but we found no organizing thrombi and no vasculopathy.

It is important to acknowledge that, despite the patient testing positive for SARS-CoV-2, there was a possibility of a co-infection with another virus, which is not uncommon in children [40].

### 4.4. Co-Pathology Analysis

Hypoxemia secondary to the patient’s COVID-19 infection is likely and could have had pathophysiologic effects on her brain via several potential mechanisms.

First and foremost, in a patient with DS, is the potential exacerbation of seizure tendency caused by hypoxia via the inhibition of GABA [41], the chief inhibitory neurotransmitter within the brain [42,43,44]. GABAergic sodium channels are already compromised genetically in Dravet syndrome [9]. The hypoxic brain with a seizure could interact with the cardiopulmonary system via ictal or post-ictal cardiac arrhythmia. Ictal bradycardia and asystole have been reported in patients with both temporal lobe and insular seizures [45]. Most instances of post-ictal asystole follow a focal onset seizure with secondary generalization. Post-ictal asystole was found to have a high mortality rate and may be responsible for many cases of SUDEP [46].

Secondly, hypoxemia itself, due to the pneumonia, must be considered to interact with the brain and heart. Chronic hypoxemia, in the absence of ischemia, does not cause neuronal necrosis [18,47,48,49]. Acute hypoxemia causes cardiac standstill [18,19] and may thereby play a role in death. Up to 5% of out-of-hospital cardiac arrests feature hypoxia secondary to pneumonia as the inciting trigger [50]. Another study identified pneumonia as the cause of cardiac arrest in 11 of 204 patients who experienced out-of-hospital cardiac arrest [51]. Since COVID-19 hypoxia presages cardiac arrest [50,51], a hypoxic cardiac arrest can be envisaged here as the cause of death, independent of the patient’s DS. In addition, an acute hypoxic event may not show immediate structural or microscopic changes in the heart despite severe hypoxia. This explains why the only cardiac abnormality noted in this case was an enlargement of the heart by 23%. This hypertrophy might not be visible microscopically. Normal adult myofibers measure ~12.3 µm in diameter [52], and a 23% increase from 10.0 µm will not be readily apparent on microscopy.

It is not possible to definitively conclude that either the seizure disorder alone or the interstitial pneumonitis alone caused the death. The fact that the child was mildly symptomatic lies within the clinical spectrum of interstitial pneumonia, with walking pneumonia and happy hypoxia being well described [53,54]. Subsequently, the mild symptoms the patient experienced days prior to death could also be due to DS. Thus, the child’s lack of grave symptoms argues for neither Dravet–SUDEP nor for pneumonia-induced hypoxic cardiac arrest [55,56]. In patients admitted to intensive care for COVID-19, one study revealed significant EEG abnormalities in 93%, with epileptiform discharges observed in 37.9% [57].

It is important that the forensic pathologist not be dogmatic in cases where either disease alone can cause death, or where co-pathology can play a shared role in causing death. Situations such as this may arise where there are two competing causes of death, and an issue of coding error arises with the International Classification of Diseases (ICD). This affects the process of death certification. One proposed solution, when two or more causally unrelated etiologies coexist, is to list the underlying cause of death as the illness that triggered the chain of events, with the immediate cause being the condition that developed immediately prior to death [58]. In this case, however, the patient’s Dravet syndrome paralleled the development of respiratory infections from infancy, further presenting challenges for completing death certification.

While a child contracting COVID-19 is noteworthy, the forensic importance of this article lies in highlighting issues with death certificate completion when two potential proximate causes of death are present, as outlined in the manuscript.

We acknowledge the limitations to this process and emphasize the need for further changes to accurately complete death certifications and ultimately reduce errors in reporting population-based mortality statistics.

## 5. Conclusions

This paper highlights a case of a 9 ½ year old patient who had two long-term health conditions: Dravet syndrome and intercurrent respiratory infections. The patient was diagnosed with Dravet syndrome during infancy and had recurrent febrile status epilepticus with prolonged hemi-convulsive seizures and ictal Todd’s paralysis, leading to developmental delay. Along with Dravet syndrome, the patient experienced recurrent respiratory infections from age 1½, with radiologic evidence of pneumonia. At age 9½, the patient experienced an unwitnessed death at home. The autopsy confirmed the presence of brain changes associated with DS, but, surprisingly, her lung tissue also revealed alveolar edema with features of proteinosis, hyaline membranes, and an infiltration of macrophages, megakaryocytes, and lymphocytes. Brain tissue showed white and gray matter edema, macrophage infiltrates in perivascular spaces, subpial gliosis, and frontal lobe gliosis in cortical layers I and II. Sections of the hippocampus showed astrogliosis in the CA1 region without neuronal loss. It is impossible to definitely conclude either DS or interstitial pneumonia as the cause of death. An interaction between hypoxic pneumonia and the profound seizure tendency in DS may be operant here. Co-pathology, as seen in this case, makes it pathophysiologically unsafe to attribute death to a single cause. Interaction between lung and brain diseases may be additive or synergistic in causing death.

## Data Availability

No new data were created or analyzed in this study.
